# Combined Effect of Race/Ethnicity and Type of Insurance on Reuse of Urgent Hospital-Based Services in Children Discharged with Asthma

**DOI:** 10.3390/children7090107

**Published:** 2020-08-20

**Authors:** Jamie M. Pinto, Sarita Wagle, Lauren J. Navallo, Anna Petrova

**Affiliations:** 1 Department of Pediatrics, Jersey Shore University Medical Center, Neptune, NJ 07753, USA; Jamie.Pinto@hackensackmeridian.org (J.M.P.); Sarita.Wagle@hackensackmeridian.org (S.W.); ljnavallo@gmail.com (L.J.N.); 2 Touchpoint Pediatrics, Chatham, NJ 07928, USA; 3 Department of Pediatrics, Rutgers Robert Wood Johnson Medical School, New Brunswick, NJ 08901, USA

**Keywords:** health disparities, social determinants, healthcare utilization, asthma, readmission

## Abstract

Asthma is a leading cause of health disparity in children. This study explores the joint effect of race/ethnicity and insurance type on risk for reuse of urgent services within a year of hospitalization. Data were collected from 604 children hospitalized with asthma between 2012 and 2015 and stratified with respect to combination of patients’ insurance status (public vs. private) and race/ethnicity (white vs. nonwhite). Highest rates for at least one emergency department (ED) revisit (49.5%, 95% CI 42.5, 56.5) and for average revisits (1.03, 95% CI 0.83, 1.22) were recorded in nonwhite children with public insurance. Adjusted models revealed higher chance for ED reuse in white as well as nonwhite children covered by public insurance. Hospitalization rate was not dependent on the combination of social determinants, but on the number of post-discharge ED revisits. The combined effect of race/ethnicity and health insurance are associated with post-discharge utilization of ED services, but not with hospital readmission.

## 1. Introduction

Asthma is a significant chronic pediatric morbidity that is also ranked as a leading cause of health disparities in minority children and in those living within urban areas and poverty [1,2,3]. Increased risk for exposure to pollutants, parental smoking, and violence-associated stress as well as unequal access to subspecialty services and poor compliance with recommended treatments are factors that could be associated with the disproportionate prevalence of asthma in children from socially disadvantaged populations [4,5,6,7,8]. Numerous publications demonstrate an association of race/ethnicity and other socioeconomic factors with asthma morbidity in childhood [8,9,10,11,12,13,14]. However, the investigation of concurrent effects from social determinants could be important to better understand the role of disparity in pediatric asthma [14]. It has been shown that Black children are at higher risk for asthma than white children, only among those from families with a low income to federal poverty level ratio [15]. Utilization of hospital-based emergency services for the treatment of asthma, including return to the emergency department (ED) after discharge, is a significant burden to the healthcare system [16] and a negative marker for quality of care [17,18] that can also be associated with patients’ social context [19,20]. In the present study, we examine an association of revisitation to the ED by children discharged with asthma with respect to their race/ethnicity combined with type of health insurance coverage. We hypothesized that among pediatric patients discharged with asthma, the frequency to return for urgent care would be highest amongst nonwhite children covered by public insurance compared to their counterparts, including nonwhite patients with private insurance and white children irrespective of type of insurance coverage. The hypothesis that was tested is based on the assumption that race/ethnicity and type of health insurance are interrelated but not completely analogous factors [3]. Moreover, we used type of insurance as a proxy for socioeconomic status for studied patients because private or public health insurance coverage reflect the parental economic and/or employment position for children in the United States [6,21]. “Public health insurance coverage for children is directly dependent on eligibility criteria, which is family income below 350% of the Federal Poverty Level in the state of New Jersey” [22]. Race/ethnicity and type of health insurance are accessible social determinants of health that could be used to predict the risk for return to urgent services for pediatric patients discharged with asthma. Findings may provide a better understanding of the implication of race/ethnicity and type of insurance on utilization of urgent services by children with asthma and, therefore, can be used in the development of preventive programs toward the reduction of disparity in childhood asthma outcomes.

## 2. Materials and Methods

This is a retrospective cohort study to determine if there is a joint effect of race/ethnicity and type of health insurance on frequency of ED revisitations with and without rehospitalizations within a year after discharge for children with asthma. The study population constituted pediatric patients hospitalized at Jersey Shore University Medical Center (JSUMC) in Neptune, New Jersey between 1 October 2012 and 30 September 2015 with asthma exacerbation. Patients were identified based on International Classification of Diseases, Ninth edition (ICD-9) diagnosis codes: 493.00, 493.01, 493.02, 493.10, 493.11, 493.12, 493.20, 493.21, 493.22, 493.80, 493.81, 493.82, 493.90, 493.91, and 493.92. The Institutional Review Board (IRB) of Meridian Health approved this study.

### 2.1. Case Verification and Data Collection

Each case was validated for eligibility based on age (from 6 months to 18 years), primary diagnosis of asthma, absence of other chronic lung diseases or congenital morbidities, as well as for availability of information regarding race/ethnicity and health insurance coverage. Demographic information (age, sex, race/ethnicity (white, Black, Hispanic, other), insurance status (private, public, no insurance/charity care)), month of admission, history of asthma (use of controller medications, previous asthma admissions, medical and family history of atopic conditions), and clinical data (fever, chest retractions, oxygen saturation) were collected. We gathered information regarding admission to the pediatric intensive care unit (PICU) and participation in our hospital-initiated Community Outreach for Asthma Care and Healthy lifestyles (COACH) program, described in a previous report [23]. Data on diagnosis and management, including use of chest radiography and oxygen supplementation, were obtained, and length of stay (LOS) was calculated. Concomitant bacterial infections were identified if any of the following diagnoses, including acute otitis media, pneumonia, urinary tract infection or other bacteria-related condition, were recognized. Chest radiographs were considered “positive” if a discrete and localized consolidation, lobar consolidation or pleural effusion was present [24]. Severity of asthma was classified based on clinical features, including pulse oximetry (PO_2_) less than 90% [25]. Fever was defined as a temperature of 100.4 F (38.0 °C) or greater [26].

### 2.2. Outcome Definition and Data Presentation

We defined reuse of urgent hospital-based services as the proportion of patients with asthma who returned for an ED visit with and without rehospitalization within 12 months of discharge. In addition, we assessed the magnitude of association between mean frequency of overall return visits after discharge and the combination of a child’s race/ethnicity as white (non-Hispanic white) or nonwhite (non-Hispanic Black, Hispanic, or other) and type of insurance as private or public, according to the United States’ Census Bureau classification [21]. Patients who received medical coverage from a charity program were combined into one category with those who were covered by public insurance because of comparable income eligibility criteria [20,27].

Each patient’s name and chart number were linked to the administrative databases of all four acute care facilities in the Meridian Health System, in order to verify number of and time to asthma-related post-discharge ED revisitation, as well as disposition of patients from the ED (discharge home or admission). We used the initial admission as the index admission to identify time (days) to subsequent ED revisits with or without readmissions during a 12 month follow-up period. Participants were included in the following groups: white patients with private insurance (Group 1), white patients with public insurance (Group 2), nonwhite patients with private insurance (Group 3), and nonwhite patients with public insurance (Group 4).

### 2.3. Statistical Analysis

Univariate analyses were conducted to compare study groups to identify differences between covariants. Continuous variables were verified for normality using the Kolmogorov–Smirnov test. We used Chi-square statistics for categorical data and analysis of variance (ANOVA) followed by the Tukey test and non-parametric statistics, such as the Kruskal–Wallis ANOVA if needed for group comparison. Categorical data are presented as a proportion (%) and continuous data as a mean with a 95% confidence interval (95% CI) or as a median and interquartile range (IQR). Regression models were constructed to identify an association of the combination of race/ethnicity and type of insurance with prevalence of at least one revisit for urgent care (logistic regression) and the total number of ED visits with or without readmission (multiple regression) occurring within a year of discharge. Factors that showed a bivariate association with the dependent variable at a level of *p* < 0.05 were entered into regression models. Identified associations are expressed with odds ratios (OR) and regression coefficient (β) with 95% CI. Data were analyzed using STATISTICA 13.3 (StatSoft Inc., Tulsa, OK, USA). All statistical tests were 2-sided with the significance level set at a *p* value of <0.05.

## 3. Results

**Figure 1:**
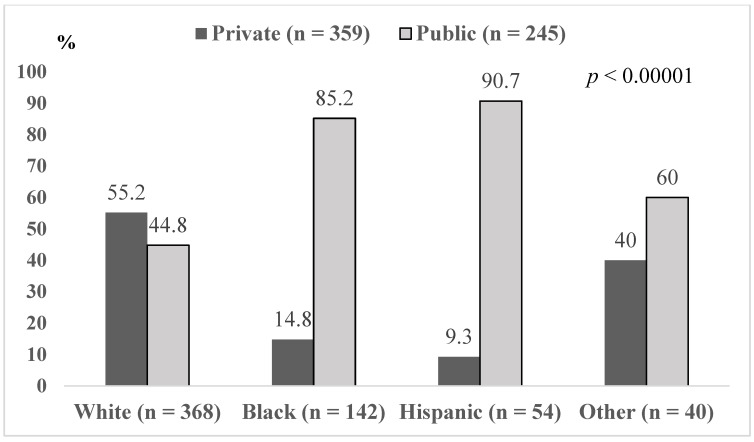
Distribution of type of health insurance coverage based on race/ethnicity.

Six hundred and forty-six children admitted with asthma exacerbation were identified. Patients aged less than six months or more than 18 years (*n* = 6), with chronic conditions other than asthma (*n* = 16), no primary diagnosis of asthma (*n* = 19), or lack of information on race/ethnicity (*n* = 1) were excluded. Among 604 patients included, 368 (60.9%) were white. Black and Hispanic patients constituted 60.2% (*n* = 142) and 22.9% (*n* = 54) of 236 nonwhite patients, respectively. Private insurance covered healthcare for 245 (40.6%) patients. Patients with public health insurance (*n* = 343) or charity coverage (*n* = 16) were assembled into a “public insurance” group. The majority of nonwhite patients had public insurance, and among white patients, public and private insurances were almost equally distributed (Figure 1). Group-based comparison of patients’ characteristics showed significant variability in patient’s age, prevalence of chest retractions, treatment with oxygen, and participation in the COACH program (Table 1). All of these covariants were taken into account in regression models designed to identify an independent association of combined race/ethnicity and type of insurance with return urgent care visits for pediatric patients discharged with asthma. Treatment with oxygen, presence of retractions, and participation in the COACH program were coded as binary variables (1 = yes and 0 = no), and age as a continuous variable.

### 3.1. Reuse of Hospital-Based Services for Children with Asthma

**Figure 2:**
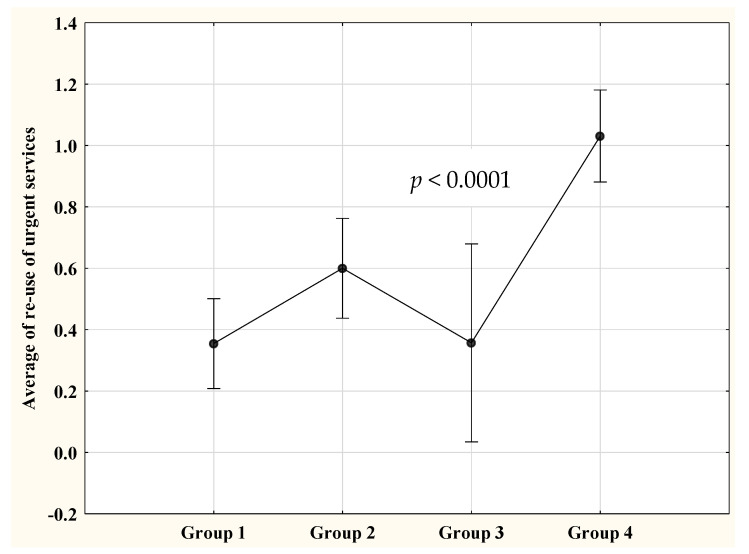
Group-based comparison of average number of revisits to the emergency department (ED) with and without hospital admission within a year after discharge. Group 1: white children with private insurance; Group 2: white children with public insurance; Group 3: nonwhite children with private insurance; Group 4: nonwhite children with public insurance; vertical bars denote 95% confidence interval.

Within a year after discharge, at least one return visit to the hospital for asthma-related care was recorded in 24.6% (95% CI 19.2, 31.0%) of 203 patients in Group 1, 33.9% (95% CI 27.2, 41.5%) of 165 patients in Group 2, 31.0% (95% CI 19.1, 46.0%) of 42 patients in Group 3, and 49.5% (95% CI 42.5, 56.5%) of 194 patients in Group 4 (*p* < 0.0001). The risk to return with asthma exacerbation between patients in Groups 1, 2, and 3 were comparable prior to and after controlling for selected covariants, but significantly lower in Group 1 compared to Group 4 both prior to and after adjustment (Table 2). Moreover, adjustment to covariants revealed significant reduction of risk for at least one urgent care revisit in patients included in Groups 2 and 3 compared to those in Group 4 (Table 2). The average number of revisits to the ED with and without hospitalization was higher in Group 4 (1.03, 95% CI 0.83, 1.22) than in Group 1 (0.35, 95% CI 0.24, 0.45), Group 2 (0.60, 95% CI 0.44, 0.76), and Group 3 (0.36, 95% CI 0.18, 0.54), *p <* 0.0001–0.02 (Figure 2). Data from controlled, multiple regression models showed that the likelihood for an increased number of ED revisits in Group 4 remained significantly higher than that of Groups 1, 2, and 3 after adjustment (Table 3). We also found that the likelihood for an increased number of ED revisits was higher for patients in Group 2 compared to those in Group 1. No association was recorded between the number of ED revisitations and the combination of race/ethnicity and type of insurance between Groups 1 and 3 nor between Groups 2 and 3. No covariant included in the models was associated with the number of ED revisits.

### 3.2. Group-Based Comparison of Hospitalization among Total Number of ED Revisitations

**Figure 3:**
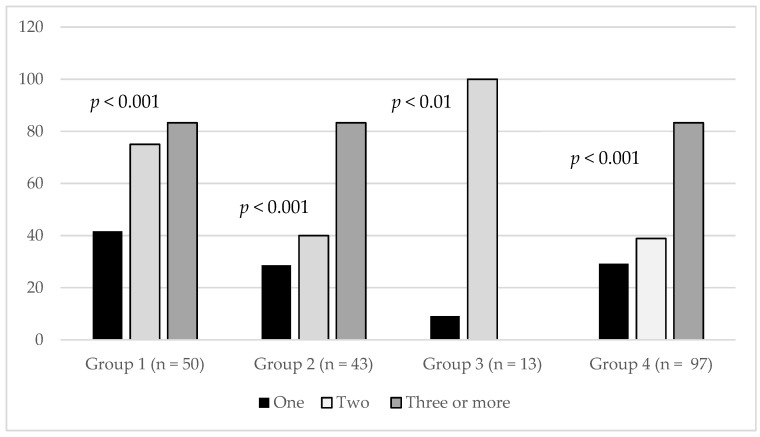
Group-based comparison of proportion of patients admitted after reevaluation in the ED and number of returns to the hospital for urgent care (one, two, three or more). Group 1: white children with private insurance; Group 2: white children with public insurance; Group 3: nonwhite children with private insurance; Group 4: nonwhite children with public insurance.

Overall, 215 patients discharged with asthma returned for evaluation in the ED, and among those, 100 (46.5%) were admitted, including 52% (26/50) in Group 1, 43.6% (24/55) in Group 2, 23.1% (3/13) in Group 3, and 47.4% (46/97) in Group 4. The difference in rate of hospitalizations between study groups did not reach statistical significance (*p* = 0.10–0.59). We found that 30.9% were hospitalized among 123 patients who visited the ED once, 48.8% among 43 patients who visited the ED twice, and 83.7% among 49 patients who visited the ED three or more times within a year after discharge (*p* < 0.0001). Comparison of the association between hospital admission and number of return visits to the ED for asthma exacerbation by study group revealed similar results (Figure 3). The association between ED reuse and risk for admission persisted in regression models that included age, participation in the COACH program, use of oxygen, and presence of retractions, both in the total sample (β 0.184, 95%CI 0.134, 0.24, *p* < 0.0001) as well as by group (Group 1 β 0.344, 95% CI 0.079, 0.874, *p* < 0.02; Group 2 β 0.712, 95% CI 0.43, 0.994, *p* < 0.001; Group 4 β 0.338, 95% CI 0.222, 0.454, *p* < 0.0001). We were not able to perform regression analysis in Group 3, because only a few patients had been admitted.

## 4. Discussion

The present study determined that nonwhite children covered by public insurance are at higher risk for reuse of urgent hospital-based services within 12 months of discharge after hospitalization for asthma than their counterparts. Data from controlled models revealed that healthcare coverage with public insurance significantly contributed to the increased frequency of post-discharge ED visits for both white and nonwhite children with asthma, with the strongest magnitude among nonwhite patients. The rate of hospitalization was not significantly dependent on the combination of tested social determinants, but on the number of post-discharge ED revisitations. Nearly one third of patients among those who revisited the ED once were admitted compared to more than 80% of patients who revisited the ED three or more times within a year of index hospitalization.

To the best of our knowledge, the joint effect of race/ethnicity and type of health insurance coverage on utilization of acute care services by children with asthma has not been studied. For the most part, studies have looked at race/ethnicity and insurance coverage for pediatric patients with asthma as relatively autonomous risk factors for utilization of ED and inpatient services amongst minority groups [8,11,28] and economically disadvantaged families [8,9,12]. Liu at al. [10] reported more than 30% higher readmission rates for pediatric patients with asthma insured by Medicaid than for children insured privately, but no significant effect of race/ethnicity was detected in adjusted regression models. According to a national survey, Black children showed higher prevalence of asthma morbidity and an increased risk for ED revisitation within one year compared to whites [2]. A large, population-based study of children with comprehensive insurance through the Military Health System showed a higher prevalence of asthma-related hospitalizations and ED visitations among pediatric patients who were Black and Hispanic compared to those who were white [6]. Moreover, a reduction in medical care for children with asthma covered by Medicaid, including less follow-up appointments and subspecialty physician visits, was found to be associated with Black and Hispanic race/ethnicity [29]. A large survey study revealed lower prevalence of childhood asthma for high socioeconomic status Black and white children with a stronger magnitude of association for white than Black families [30].

Few studies have tried to explain the interface of cultural and economic status on outcomes for childhood asthma. It has been shown that the relationship between risk for hospitalization and exposure to nitrogen dioxide, a marker of pollution was modified by race/ethnicity and insurance status [29]. Consequently, the risk for asthma exacerbation was disproportionate for minority children with or without health insurance coverage [31]. However, a survey of children with asthma did not find an association with insurance status but did link nonwhite race/ethnicity with use of urgent care services within one year [32]. Chang et al. [33] reported a stronger association between repeat hospital visits and residence near heavily trafficked streets for children who were uninsured or covered by Medicaid than for privately insured pediatric patients with asthma.

This study has several limitations. One is that the number of nonwhite patients with private health insurance is disproportionate to other study groups; however, this reflects the disparity in type of medical insurance coverage for children in the United States [34]. Another that should be considered is inclusion in this study of only race/ethnicity and type of health insurance because only these social determinants of health are available to medical professionals at point of care and, therefore, for identification of patients prior to discharge for targeted interventions. It is possible that implicit bias could affect the risk for return visits to the hospital for asthma exacerbation among nonwhite children secondary to reduced prescriptions for asthma controller medications by pediatricians, although we recorded no difference in use of controllers between the study groups (Table 1). However, we believe that non-compliance with use of controller medications may increase the risk for exacerbation of asthma in children irrespective to their cultural background. Conducting a prospective study is needed to obtain information on both outpatient medication prescriptions and compliance with treatment recommendations for children with asthma. An additional limitation is that retrospective study design could affect data quality. Case verification, use of a standardized data collection tool, and controlled regression models may reduce the risk of biases (i.e., selection and misclassification) and role of confounders associated with retrospective design. Furthermore, the study is single-center, which may limit applicability of results in other settings.

## 5. Conclusions

The frequency of return visits for urgent care in nonwhite patients with public insurance within a year after discharge for asthma was higher than in all other study participants. Furthermore, controlled models revealed an association between healthcare coverage with public insurance and recurrence of ED visits not only for nonwhite children, but also for white participants, although at a lower magnitude than for their nonwhite counterparts. We found that the risk for rehospitalization was unrelated to the combination of race/ethnicity and type of insurance; however, it was substantially dependent on repeat use of ED services. Despite limitations, study findings could be used to develop or enhance programs designed to improve asthma morbidity, by targeting pediatric patients at increased risk for reutilization of urgent care services prior to hospital discharge. Further research is needed, including multicenter studies with a larger sample to add knowledge regarding the role of social determinants that can be easily accessed by medical professionals at point of care, on asthma outcomes throughout childhood.

## Figures and Tables

**Table 1:** Comparison of patients’ demographic and clinical characteristics by Group †.

Characteristics ‡	Group 1 *n* = 203	Group 2 *n* = 165	Group 3 *n* = 42	Group 4 *n* = 194	*p* Value
Age (years)	5 (3–8)	4 (2–7)	7 (2–11)	4 (1.8–7)	0.01
Male sex	129 (63.6)	97 (58.8)	26 (61.9)	118 (60.8)	0.82
Prior asthma admission (s)	77 (38.1)	71 (43.3)	19 (45.2)	98 (50.8)	0.09
Prior PICU asthma admission(s)	23 (12.3)	23 (15.2)	6 (15.8)	28 (16.1)	0.75
Use of controller medication(s)	72 (35.5)	59 (36.0)	17 (40.5)	76 (39.2)	0.82
Winter season at admission	49 (24.1)	48 (29.1)	11 (26.2)	46 (23.7)	0.98
PICU hospitalization	61 (30.2)	48 (29.1)	11 (26.2)	53 (27.5)	0.91
Fever at presentation	94 (46.3)	89 (53.9)	18 (42.9)	94 (48.5)	0.41
Chest retractions	130 (64.4)	111 (67.3)	21 (50.0)	146 (75.3)	<001
Oxygen supplementation	111 (55.0)	111 (67.3)	16 (38.1)	95 (49.2)	<0.001
PO2 < 90% on presentation	30 (14.9)	29 (17.7)	4 (9.8)	20 (10.3)	0.18
Antibiotic treatment	112 (55.5)	86 (52.1)	21 (51.2)	106 (54.9)	0.89
Bacterial infection	33 (16.3)	21 (12.7)	5 (11.9)	28 (14.5)	0.76
Chest X-ray	190 (94.1)	158 (95.8)	40 (95.2)	184 (94.9)	0.91
Positive Chest X-ray	23 (12.0)	15 (9.5)	4 (12.5)	15 (8.2)	0.59
COACH participation	39 (19.2)	37 (22.4)	7 (16.7)	70 (36.1)	<0.001
LOS (hours)	51 (35–89.8)	64.2 (42.5–96.8)	52.8 (33.5–110)	65.5 (41.1–110.8)	0.11

† Group 1: white children with private insurance; Group 2: white children with public insurance; Group 3: nonwhite children with private insurance; Group 4: nonwhite children with public insurance; ‡ data presented as median with interquartile range (IQR) and number with percentage (%).

**Table 2:** Association of the combinations of race/ethnicity and type of insurance with at least one reuse of urgent care services within a year after discharge †.

Group ‡ (Code)	Group 2 (1)		Group 3 (1)		Group 4 (1)	
	OR_crude_	OR_adjusted_	OR_crude_	OR_adjusted_	OR_crude_	OR_adjusted_
Group 1 (0)	0.88 (0.64, 1.21)	0.81 (0.64, 1.02)	0.92 0.55,1.54)	0.85 (0.59, 1.23)	0.67 ** (0.49, 0.92)	0.57 ** (0.46, 0.70)
Group 2 (0)			1.05 (0.61, 1.78)	1.07 (0.74, 1.54)	0.77 (0.54, 1.08)	0.71 *** (0.57, 0.88)
Group 3 (0)					0.73 (0.43, 1.25)	0.66 * (0.47, 0.96)

† Data presented as odds ratios (OR_crude_ and OR_adjusted_) with 95% confidence interval (95% CI); ‡ Group 1: white children with private insurance; Group 2: white children with public insurance; Group 3: nonwhite children with private insurance; Group 4: nonwhite children with public insurance; in each group-based model, the group number was coded as 0 and 1; 0 was applied to the group with the lower number and 1 to the group with the higher number; * *p* < 0.03, ** *p* < 0.02, *** *p* < 0.01.

**Table 3:** Multiple regression models (β and 95%CI) to identify an association of the combination of race/ethnicity and type of insurance with repeat ED visits.

Group † (Code) ‡	Group 2 (1)	Group 3 (1)	Group 4 (1)
Group 1 (0)	0.231 (0.055, 0.407) *	0.011 (−0.011, 0.133)	0.223 (0.148, 0.298) ***
Group 2 (0)		−0.235 (−0.578, 0.108)	0.221 (0.088, 0.354) **
Group 3 (0)			0.664 (0.209, 1.119) **

† Group 1: white children with private insurance; Group 2: white children with public insurance; Group 3: nonwhite children with private insurance; Group 4: nonwhite children with public insurance; ‡ in each group-based model, the group number was coded as 0 and 1; 0 was applied to the group with the lower number and 1 to the group with the higher number; * *p* < 0.03, ** *p* < 0.01, *** *p* < 0.001.
